# Inactivation of human and avian influenza viruses by potassium oleate of natural soap component through exothermic interaction

**DOI:** 10.1371/journal.pone.0204908

**Published:** 2018-09-27

**Authors:** Takayoshi Kawahara, Isamu Akiba, Megumi Sakou, Takemasa Sakaguchi, Hatsumi Taniguchi

**Affiliations:** 1 Department of Microbiology, University of Occupational and Environmental Health, Kitakyushu, Fukuoka, Japan; 2 Research and Development Department, Shabondama Soap Co., Ltd., Kitakyushu, Fukuoka, Japan; 3 Department of Chemistry and Biochemistry, The University of Kitakyushu, Kitakyushu, Fukuoka, Japan; 4 Department of Virology, Graduate School of Biomedical and Health Sciences, Hiroshima University, Hiroshima, Japan; Northeastern University, UNITED STATES

## Abstract

An influenza epidemic is still a problem despite the development of vaccines and anti-influenza drugs. Preventive measures such as handwashing are fundamental and important for counteracting influenza virus infection. In this study, we clarified the anti-influenza virus effects of surfactants, which are the main components of hand soaps for hand washing: potassium oleate (C18:1), sodium laureth sulfate (LES) and sodium lauryl sulfate (SDS). For a human influenza virus strain (H3N2), C18:1 reduced the infectivity by 4 logs or more, whereas LES and SDS reduced the infectivity by 1 log or less. Similar results were obtained when an avian influenza virus strain (H5N3) was used. The interaction between the surfactant and virus was then investigated by isothermal titration calorimetry. The LES-virus system showed a positive value of enthalpy changes (ΔH), meaning an exothermic interaction that indicated a hydrophobic interaction. In contrast, both the C18:1-virus system and the SDS-virus system showed negative values of ΔH, meaning an endothermic interaction that indicated an electrical interaction. The ΔH value of the C18:1-virus system was much higher than that of the SDS-virus system. A mixture of C18:1 and HA proteins similarly showed negative values of ΔH. These results indicate that influenza virus inactivation by a hydrophobic interaction of a surfactant with the viral envelope is insufficient to prevent infection, whereas inactivation by an electrical interaction of a surfactant with HA proteins is sufficient to prevent influenza virus infection.

## Introduction

Influenza virus, which belongs to the family *Orthomyxoviridae*, has a virus particle (virion) of ca. 100 nm in diameter. The virion is covered with an envelope composed of a lipid bilayer and inserted viral spike proteins, hemagglutinin (HA), neuraminidase (NA) and M2 ion channel proteins. One influenza virus particle is estimated to contain roughly 700 HA proteins, 200 NA proteins and 20 M2 proteins [1].

Influenza virus causes an outbreak every year, disturbing social activities at schools and workplaces and increasing medical expenses [2]. Influenza is thought to be the cause of numerous deaths estimated as excess mortality, especially for elderly persons, chronically ill patients and children [3]. Furthermore, there is always the risk that a new strain of influenza virus will emerge and cause a pandemic. The emergence and outbreak of the pandemic virus 2009 (H1N1) is still fresh in the minds of many, and there are growing concerns that epidemics of avian influenza virus H5N1 or H7N9 subtype may occur among humans in the future [4, 5].

Influenza virus infection can be prevented by vaccines and can be treated with anti-influenza drugs. However, these measures might be ineffective due to antigenic changes or drug resistance of influenza viruses. Preventive measures such as handwashing and wearing masks are fundamental for counteracting influenza virus infection.

A hand soap used for handwashing contains a surfactant as a basic ingredient. Synthetic surfactants such as sodium laureth sulfate (LES) and sodium lauryl sulfate (SDS) are used for hand soaps [6]. A surfactant is an important component that contributes to detergency and foaming, which determine the basic performance of a hand soap. Soap, a salt of fatty acid, is usually generated from natural oils and fats and is also utilized for hand soap. Although it is generally accepted that surfactants dissolve the lipid bilayer membrane of influenza virions [7], the precise mechanism of this action is unknown. Differences in effects among surfactants are also unknown.

The aim of the present study was to clarify the anti-influenza virus effects of representative surfactants, LES, SDS and potassium oleate (C18:1), that are generally used in hand soap and to elucidate the mechanism of action in order to provide insights for the development of a new hand soap.

## Materials and methods

### Cells and viruses

MDCK(+) cells, canine renal epithelial cells, were purchased from ATCC as MDCK cells (NBL-2: ATCC CCL-34) through Dainippon Pharmaceutical Co. (Tokyo, Japan) as described by Noma et al. [8], and the cells were propagated in minimum essential medium (MEM; Invitrogen, Carlsbad, CA) supplemented with 10% fetal calf serum (FCS; PAA Laboratories, Pasching, Austria), 100 units/ml penicillin G (Meiji Seika Pharma, Tokyo, Japan) and 100 μg/ml streptomycin (Meiji Seika Pharma) at 37°C in the presence of 5% CO_2_ gas.

Ten-day-old chicken embryonated eggs were purchased from Hiroshima Experimental Animals Corp. (Hiroshima, Japan). The eggs were not in a specific-pathogen-free grade but maintained in a bioclean condition.

Human influenza virus A/Udorn/72 (H3N2) and avian influenza virus A/whistling swan/Shimane/499/83 (H5N3) were provided by Dr. R. A. Lamb (Northwestern University, IL) and Dr. K. Otsuki (Tottori University, Japan), respectively, and propagated in embryonated chicken eggs.

### Reagents

Sodium dodecyl sulfate (SDS), sodium laureth sulfate (LES) and oleic acid were purchased from Wako Pure Chemical Industries (Osaka, Japan), NOF Corporation (Tokyo, Japan) and Tokyo Chemical Industry (Tokyo, Japan), respectively. Potassium hydroxide was purchased from Wako Pure Chemical Industries. All reagents were used without further purification.

An influenza HA vaccine for the 2013–2014 season (manufacturing number HA130A), containing the HA proteins of A/California/7/2009(H1N1) pdm09, A/Texas/50/2012(H3N2) and B/Massachusetts/2/2012, was purchased from Biken Corporation Ltd. (Osaka, Japan). The vaccine was a split vaccine made from purified virus particles and contained more than 90 µg/ml of the HA proteins from the three viruses.

### Preparation of an aqueous surfactant solution

SDS and LES were dissolved in deionized water at 0.35 mmol/l. To enhance homogeneous dispersion, the solutions were heated at 40°C for 1 h. Oleic acid was dissolved in deionized water at 0.35 mmol/l, and then an equimolar amount of potassium hydroxide was added and the solution was heated at 75°C for 1 h for conversion to potassium oleate (C18:1). Finally, the pH of the aqueous C18:1 solution was adjusted to 10.3 by adding potassium hydroxide.

### Anti-virus assay

An anti-virus assay for influenza virus was performed as described previously [9]. Briefly, 10, 100, and 1000-times-diluted surfactant solutions (35 mmol/l, 3.5 mmol/l, and 0.35 mmol/l, respectively) were mixed with a virus solution in a ratio of 9:1, and after 3-min incubation at room temperature, each solution was 10-fold serially diluted with Dulbecco’s phosphate-buffered saline (PBS). MDCK(+) cells in a 96-well plate were inoculated with 50 μl of a diluted virus solution in quadruplicate. After 1-h adsorption, the inoculum was removed and cells were incubated in 100 μl/well of Dulbecco’s MEM (DMEM) supplemented with penicillin G, streptomycin (Invitrogen) and 20 μg/ml crude trypsin (Merck, Darmstadt, Germany). Sterile water was used instead of surfactant solution as a mock-treated control. When cytopathic effects (CPEs) had fully developed after several days, the cells were fixed with ethanol and acetate (5:1) and further stained with 0.5% amido black 10B in 45% ethanol and 10% acetate. The 50% endpoint of virus infection was determined by the Behrens-Karber method, and the 50% tissue culture infectious dose (TCID_50_) was calculated. The anti-virus effect was estimated by comparing surfactant-treated infectivity with mock-treated infectivity.

Influenza virus particles were purified as described previously [9]. Briefly, 10-day-old embryonated chicken eggs were inoculated with influenza virus and incubated for 48 h at 37°C and then euthanized by keeping at 4°C overnight. The infected allantoic fluids were collected and centrifuged at 24000 rpm for 1 h in a Beckman SW28 rotor. The virus pellets were suspended in PBS and centrifuged at 24000 rpm for 2 h through a 20–50% continuous sucrose gradient in a SW28 rotor. The virus band was then collected, dialyzed against PBS, and concentrated by using an Amicon Ultra-15 filter unit (Merck), followed by inactivation with UV irradiation. The protein profile of the virus particles was checked by SDS-polyacrylamide gel electrophoresis and Coomassie brilliant blue staining.

### Isothermal titration calorimetry (ITC)

ITC experiments were performed at 25°C using a VP-ITC MicroCal microcalorimeter (Northampton, MA). An aqueous surfactant solution (1.75×10^−1^ mmol/l) was maintained in the ITC cell (1.4 ml) at 25°C with stirring at 300 rpm. Aliquots of virus solution (total protein = 1.5 mg/ml) were injected into the ITC cell using a microsyringe. The volume of each injection was 5 μl. The duration of each injection was 14 s, and there was an interval of 250 s to allow for equilibration correction. The heat of dilution was subtracted even though its contribution to total heat was negligibly small. The enthalpy changes (ΔH) due to interaction were determined from the titration data on the basis of the amount of surfactant molecules.

### Statistical analysis

All data analyses were repeated at least three times, and the *p* value was determined by Student’s t test. *p* <0.05 was considered statistically significant.

## Results

### Inactivation of influenza virus by surfactants

**Fig 1:**
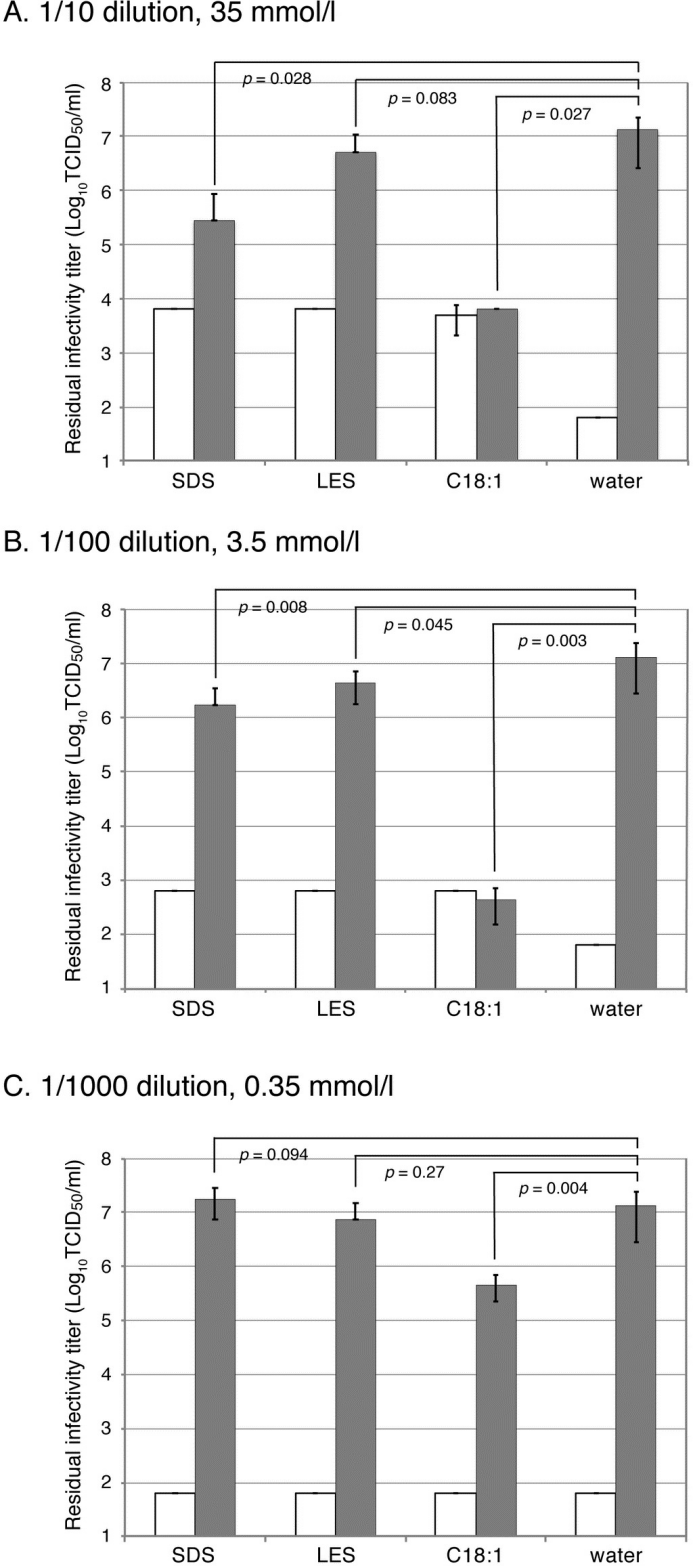
Anti-virus effects of surfactants on influenza virus A/Udorn/72 (H3N2). Sodium dodecyl sulfate (SDS), sodium laureth sulfate (LES) and potassium oleate (C18:1) were used as surfactants at concentrations of 35 (A), 3.5 (B) and 0.35 (C) mmol/l. One volume of the solution containing influenza virus A/Udorn/72 (H3N2) was mixed with nine volumes of the surfactant solution and further incubated for 3 min at room temperature, followed by measurement of the remaining infectivity. Gray bars show viral infectivity and white bars show the cytotoxicity of the surfactants without the virus. Error bars indicate standard deviation, and *p* values are indicated in the figure.

Human influenza virus H3N2 was incubated with different concentrations of the surfactants LES, SDS and C18:1. The residual infectivity values of the viruses are shown in Fig 1. At a concentration of 3.5 mmol/l (Fig 1B), C18:1 significantly reduced the infectivity by more than 4 logs (*p* = 0.003), whereas SDS and LES reduced the infectivity by 1 log or less, although the reductions were statistically significant. In the case of 10-fold concentrated surfactants (Fig 1A), similar results were obtained, although the difference appeared to be less due to the high background caused by the high cytotoxicity of the surfactants to cultured cells. In the case of 10-fold diluted surfactants (Fig 1C), the effect of C18:1 was less but only the difference in C18:1 was statistically significant among the three surfactants.

**Fig 2:**
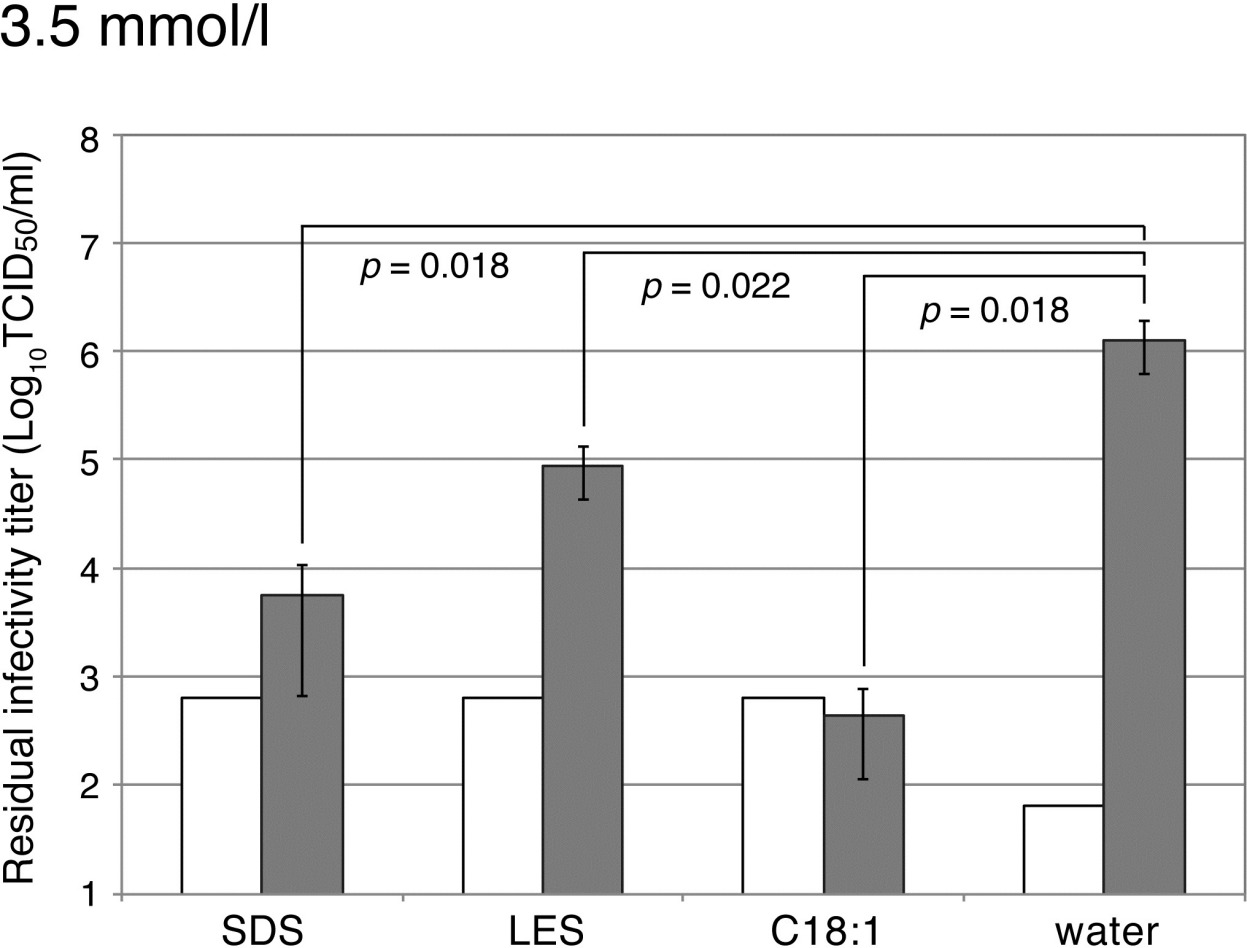
Anti-virus effects of surfactants on avian influenza virus A/swan/Shimane/499/83 (H5N3). Sodium dodecyl sulfate (SDS), sodium laureth sulfate (LES) and potassium oleate (C18:1) were used as surfactants at the concentration of 3.5 mmol/l. One volume of the solution containing avian influenza virus A/swan/Shimane/499/83 (H5N3) was mixed with nine volumes of the surfactant solution and further incubated for 3 min at room temperature. Gray bars show viral infectivity and white bars show the cytotoxicity of the surfactants without the virus. Error bars indicate standard deviation, and *p* values are indicated in the figure.

When avian influenza virus H5N3 was used as a test virus, the infectivity was reduced by more than 3 logs with C18:1, while it was reduced by ca. 2 logs with SDS and by ca. 1 log with LES (Fig 2). C18:1 was thus found to be a surfactant with stronger anti-virus effects than those of SDS and LES against both H3N2 and H5N3 viruses.

### Thermal interaction between influenza virus and surfactant

**Fig 3:**
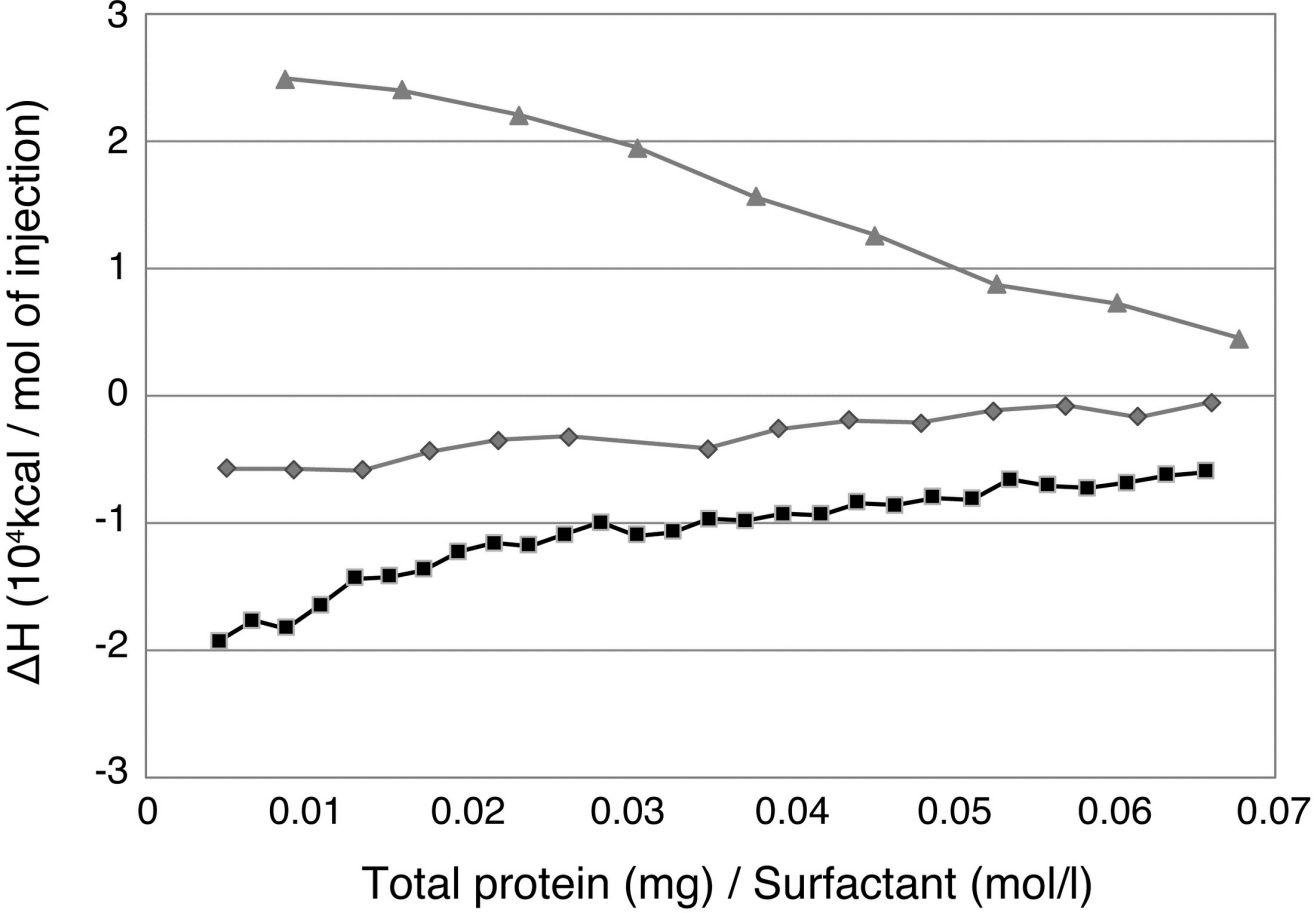
Thermodynamic profiles for ITC measurements between the virus and a surfactant. Virus particles of A/Udorn/72 (H3N2) were purified and concentrated, and the virus solution (total protein, 1.5 mg/ml) was injected into a surfactant solution (17.5 mmol/l). ▲:LES, ◆:SDS, ◼:C18:1.

To clarify the reason for the differences in the anti-virus effects of the surfactants, we focused on the interaction between the virus and each surfactant. The ΔH values by mixing the virus with surfactants in aqueous media obtained from ITC measurements are shown in Fig 3. Negative and positive ΔH values indicate exothermic and endothermic interactions, respectively. An exothermic interaction corresponds to an attractive interaction such as an electrostatic interaction or hydrogen bonding, whereas an endothermic interaction corresponds to an interaction with an increment of entropy such as a hydrophobic interaction. The LES-virus system showed positive value of ΔH, indicating a hydrophobic interaction between LES and virus particles. The hydrophobic interaction in the mixtures of LES and the virus means fusion of the viral envelope composed of a lipid bilayer with the hydrophobic tail of LES [10]. On the other hand, both the C18:1-virus and SDS-virus systems showed negative values of ΔH, indicating an attractive interaction between surfactant molecules and virus particles. The absolute value of ΔH of the C18:1-virus system was much higher than that of the SDS-virus system, indicating that the attractive force between C18:1 and the virus was much stronger than that between SDS and the virus.

## Discussion

It is generally believed that the mechanism of influenza virus inactivation by a surfactant is fusion with the envelope membrane by a hydrophobic interaction [11]. This is because the influenza virus surface is covered by a lipid bilayer of the envelope membrane that interacts readily with the hydrophobic group of a surfactant. Since the LES-virus system showed an endothermic interaction, a hydrophobic interaction is dominant between LES and the influenza virus. Among the three surfactants used in this study, LES showed the weakest anti-virus effect. Therefore, the efficiency of inactivation of an influenza virus by fusion of a surfactant with the envelope by a hydrophobic interaction is insufficient to prevent infection of the influenza virus. On the other hand, both the SDS-virus and C18:1-virus systems showed exothermic interactions, suggesting that electrical interactions occur between these surfactants and the influenza virus. As is well known, SDS and C18:1 are anionic surfactants. In contrast, the envelope membrane of an influenza virus is negatively charged like the lipid membrane of a cell. The hydrophilic groups of SDS and C18:1 and the envelope membrane of influenza virus are both negatively charged and therefore do not undergo an electrical interaction. Then, which portion of the influenza virus interacted with SDS and C18:1? An influenza virus has spike proteins, including HA, NA and M2, protruding from the envelope. Among these, the HA protein is the major population and tends to be positively charged [12, 13]. It is therefore considered that the hydrophilic groups of SDS and C18:1 interacted electrostatically with the HA protein to be adsorbed to the influenza virus and inactivated the virus. Actually, the interaction between C18:1 and HA has been shown to be an attractive interaction (S1 Fig). Furthermore, since the absolute value of ΔH of the C18:1-virus is much higher than that of the SDS-virus, the electrostatic interaction between C18:1 and HA is much stronger than that of the SDS-virus. C18:1 showed a stronger anti-virus effect than that of SDS. SDS has sulfonic acid as the hydrophilic group and has a large ionic radius. On the other hand, C18:1 has carboxylic acid as the hydrophilic group and has a smaller ionic radius than that of SDS. This means that the repulsion of C18:1 against the envelope membrane of the influenza virus is weaker than that of SDS. Accordingly, C18:1 could approach the virus surface more easily in comparison with SDS. It is therefore considered that electrostatic interaction could interact electrically with HA more readily than could SDS. C18:1 is thus thought to exhibit a stronger exothermic interaction and consequently show a stronger anti-virus effect than that of SDS.

Thus, the exothermic interaction between C18:1 and spikes of the influenza virus is considered to mainly contribute to the anti-virus effect. Since HA acts at the initial stage of influenza virus propagation, it is possible that inactivation of a virus and inhibition of its propagation can be achieved by using soap. In addition, an exothermic interaction is an electrical interaction and is therefore an extremely fast reaction. That is, C18:1 quickly interacts with an influenza virus, and a virus might therefore be inactivated within a short time in performing handwashing. A hand soap that uses C18:1 as a main component might therefore be an effective hand soap for preventing infection of an influenza virus.

It is also known that the positive charge of HA has been increasing in its evolutionary process [12]. It is believed that this enables smooth adsorption onto human cells, which are negatively charged [13]. C18:1 interacts with the positive charge of HA and therefore may exothermically interact more strongly as an influenza virus evolves. C18:1 may therefore be used to produce a hand soap that is effective as a countermeasure against influenza in the future.

## Supporting information

S1 Fig Thermodynamic profiles for ITC measurements between HA and C18:1. A commercial HA vaccine, containing more than 0.09 mg/ml of the HA proteins of A/California/7/2009(H1N1) pdm09, A/Texas/50/2012(H3N2) and B/Massachusetts/ 2/2012, was injected into C18:1 solution (17.5 mmol/l). The C18:1-HA system showed a negative value of ΔH, indicating an attractive interaction between C18:1 and HA.
(TIF)
