# Supplementary figures and images for: Inactivation of human and avian influenza viruses by potassium oleate of natural soap component through exothermic interaction
Source: PLoS One. 2018 Sep 27;13(9):e0204908. doi: 10.1371/journal.pone.0204908 (PMC6160177; doi:10.1371/journal.pone.0204908)

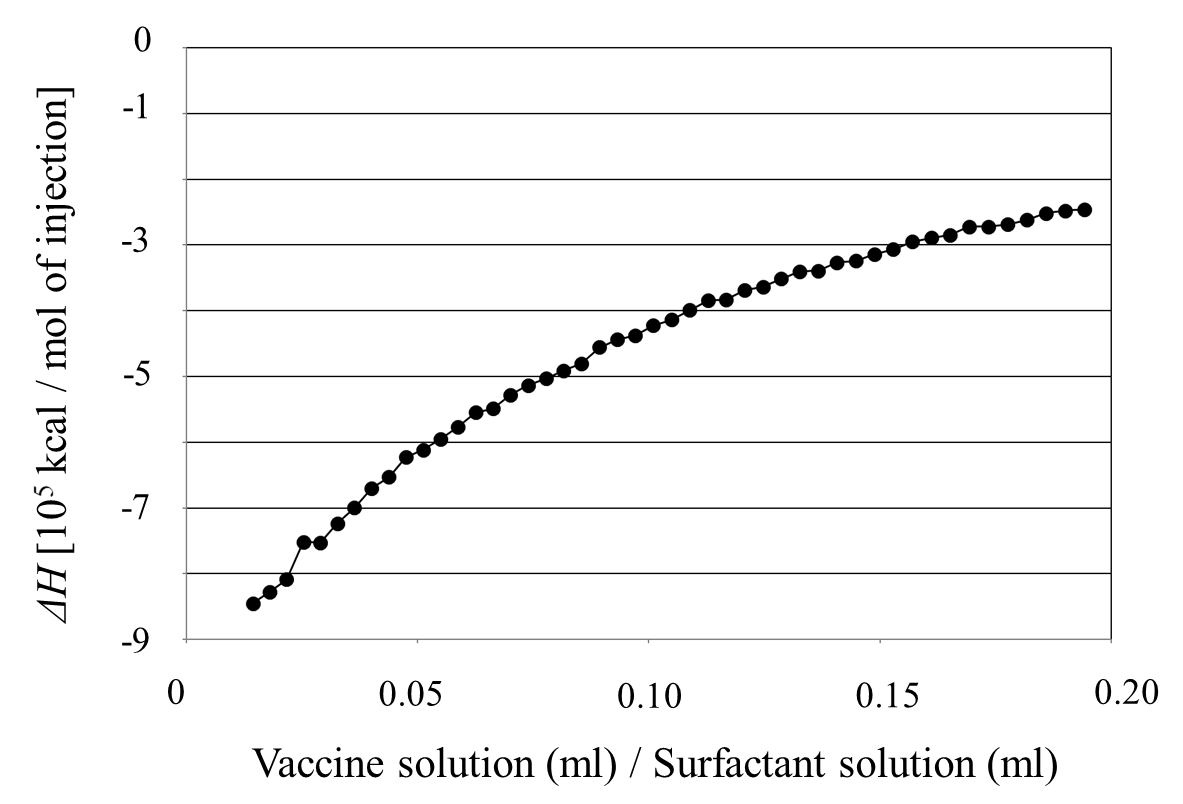

Supplement: S1 Fig — A commercial HA vaccine, containing more than 0.09 mg/ml of the HA proteins of A/California/7/2009(H1N1) pdm09, A/Texas/50/2012(H3N2) and B/Massachusetts/ 2/2012, was injected into C18:1 solution (17.5 mmol/l). The C18:1-HA system showed a negative value of ΔH, indicating an attractive interaction between C18:1 and HA. (TIF) [file pone.0204908.s001.tif]
